# Age‐related normal limits for spatial vision

**DOI:** 10.1111/opo.13037

**Published:** 2022-08-18

**Authors:** Arjan Keuken, Ahalya Subramanian, Sigrid Mueller‐Schotte, John L. Barbur

**Affiliations:** ^1^ Applied Vision Research Centre, The Henry Wellcome Laboratories for Vision Science City, University of London London UK; ^2^ Department of Optometry University of Applied Sciences Utrecht The Netherlands; ^3^ Department Technology for Healthcare Innovations University of Applied Sciences Utrecht The Netherlands

**Keywords:** contrast thresholds, functional contrast sensitivity, mesopic vision, normal age limits, photopic vision, visual acuity

## Abstract

**Purpose:**

To establish age‐related, normal limits of monocular and binocular spatial vision under photopic and mesopic conditions.

**Methods:**

Photopic and mesopic visual acuity (VA) and contrast thresholds (CTs) were measured with both positive and negative contrast optotypes under binocular and monocular viewing conditions using the Acuity‐*Plus* (AP) test. The experiments were carried out on participants (age range from 10 to 86 years), who met pre‐established, normal sight criteria. Mean and ± 2.5σ limits were calculated within each 5‐year subgroup. A biologically meaningful model was then fitted to predict mean values and upper and lower threshold limits for VA and CT as a function of age. The best‐fit model parameters describe normal aging of spatial vision for each of the 16 experimental conditions investigated.

**Results:**

Out of the 382 participants recruited for this study, 285 participants passed the selection criteria for normal aging. Log transforms were applied to ensure approximate normal distributions. Outliers were also removed for each of the 16 stimulus conditions investigated based on the ±2.5σ limit criterion. VA, CTs and the overall variability were found to be age‐invariant up to ~50 years in the photopic condition. A lower, age‐invariant limit of ~30 years was more appropriate for the mesopic range with a gradual, but accelerating increase in both mean thresholds and intersubject variability above this age. Binocular thresholds were smaller and much less variable when compared to the thresholds measured in either eye. Results with negative contrast optotypes were significantly better than the corresponding results measured with positive contrast (*p* < 0.004).

**Conclusions:**

This project has established the expected age limits of spatial vision for monocular and binocular viewing under photopic and high mesopic lighting with both positive and negative contrast optotypes using a single test, which can be implemented either in the clinic or in an occupational setting.

**Key points**

- Boundaries for healthy aging of spatial vision have been established under both photopic (daylight) and low mesopic (twilight) lighting for 16 different stimulus conditions.
- Equations that describe normal aging limits and full measures of variability in spatial vision have been produced using monocular and binocular thresholds measured with increments and decrements in luminance.
- The normal aging limits for spatial vision obtained in this study with standardized stimulus conditions have immediate use in the clinic and visually demanding occupational environments.

## INTRODUCTION

Photopic visual acuity (VA) measurements are common in clinical practice and determine the patient's ability to resolve fine detail in high contrast.^1^ The most common VA tests employ high light levels when the pupil size and higher order aberrations are small and retinal sensitivity to contrast is high. The results of the tests are often not representative of typical working environments but are easy and simple to carry out and in general extremely useful. However, VA tests are not sufficiently sensitive to measure small changes in visual performance caused by increased higher order aberrations and scattered light.^2^ They also fail to detect small changes in the early stages of ocular disease such as diabetic retinopathy, glaucoma and age‐related macular degeneration.^3^, ^4^, ^5^, ^6^ Some of these shortcomings may be overcome by also measuring VA at a lower light level in the high mesopic range.^7^ In addition to VA, contrast sensitivity (CS), defined as the reciprocal of stimulus contrast at a threshold, also yields useful information on the kind of spatial vision one can achieve. Full measurements of CS with sinusoidal gratings as a function of spatial frequency and visual field size yield a great deal of useful information but take a long time to carry out, and the results depend on the mode of stimulus presentation (e.g., briefly presented or drifting gratings) and the subject's threshold criterion (e.g., just noticeable bright or dark bars, motion direction, local flicker or just anything different to a uniform field).^8^, ^9^ These disadvantages, particularly the long testing times, make full CS tests unattractive for use in the clinic. A compromise is to use constant size optotypes of varying luminance contrast and to measure the smallest contrast needed to just name the letters correctly.^10^ The choice of optotype and optimum size have evolved over several years. Landolt rings with an outer diameter of 15 min of arc are frequently employed in such tests, largely because a gap size of 3 min arc is considered functionally important in almost every occupation, and at the same time is large enough to ensure that the majority of patients can carry out the task. A 15 min arc optotype is less affected by small residual refractive errors, large higher order aberrations and scattered light. A Landolt C optotype has additional advantages in that a four‐alternative, forced response procedure can be implemented in a two‐down, one‐up staircase,^11^ with variable step sizes, which result in low chance probability (i.e., 1/16). This test procedure is statistically efficient and its implementation on calibrated visual displays that allow for the use of both luminance increments and decrements make this measurement of contrast thresholds (CTs) appropriate for use in both occupations and in the clinic.^12^, ^13^ Furthermore, CTs measured this way require the correct detection of the position of the gap in the Landolt ring and not the much lower contrast threshold needed to just detect the presence of the ring. As a result, the reciprocal of the CTs measured in this study yield much lower CS values. In order to distinguish the absolute measures of CS using sinusoidal gratings from functional tests that also measure CTs but require either the naming of a letter or the correct localization of the gap in a Landolt ring, the reciprocal of the measured contrast threshold is described as functional contrast sensitivity (FCS). Previous studies have found a good correlation between the measurement of CTs and the level of comfort and visual performance one can achieve in normal daily tasks.^1^, ^14^, ^15^, ^16^, ^17^ CS and FCS have been shown to be more sensitive to changes in retinal image quality caused by the optics of the eye when compared to VA, but the loss in both VA and sensitivity to contrast can also be attributed to neural changes with increasing age caused by reduction in cone sensitivities, loss of photoreceptors,^18^ reduced photon absorption efficiency in cones^19^ and/or neural changes in the retina caused by normal aging and/or disease.^1^, ^4^, ^13^, ^20^, ^21^, ^22^, ^23^, ^24^, ^25^, ^26^

The rate of loss of visual sensitivity with decreasing retinal illuminance is also indicative of age‐related changes and/or the presence of early‐stage retinal disease.^13^ In addition to measurements in the photopic range, it is therefore of interest to measure VA and sensitivity to contrast in the upper mesopic range when adequate spatial vision is maintained despite increased within and intersubject variability.^27^, ^28^ There are also other reasons why assessment of spatial vision in the mesopic range is important. Pupil size affects higher order aberrations and retinal illuminance and can also alter the effects of scattered light when scattering is nonuniform over the pupil.^27^

Normal aging affects both VA and CS.^13^, ^20^, ^29^, ^30^, ^31^, ^32^, ^33^, ^34^, ^35^, ^36^, ^37^, ^38^, ^39^, ^40^, ^41^, ^42^ Previous studies found a decline in photopic VA and CS beyond ~60 years of age.^29^, ^36^, ^37^ Under mesopic conditions this decline starts at an earlier age,^20^, ^35^, ^43^ as a result of pupil miosis, increased light scatter and absorption of light by the lens and potentially also as a result of changes in the retina and visual pathways.^32^, ^36^, ^44^, ^45^ The age‐related decline in rod photoreceptor density is well documented,^44^ and postreceptoral changes, such as loss of ganglion cells and in particular damage to their retinal axons contribute to the worsening of spatial vision in normal aging.^46^ Both photopic and mesopic CS are more sensitive in the early detection of vision changes in retinal disease when compared with VA alone.^1^, ^4^, ^20^, ^21^, ^22^, ^23^, ^24^, ^25^, ^26^ It is well established that normal aging affects VA and CS in photopic and mesopic light levels,^13^, ^20^, ^29^, ^30^, ^31^, ^32^, ^33^, ^34^, ^35^, ^36^, ^37^, ^38^, ^39^, ^40^, ^41^, ^42^ but the decline in CS is more pronounced when compared with VA.^36^, ^41^ Knowledge of the effects of normal aging on spatial vision makes it possible to detect abnormal changes that can be attributed either to the optics of the eye or to early‐stage retinal disease. The availability of normal age limits for VA and CTs, in both photopic and mesopic conditions, may make it possible to separate changes caused by normal aging from those caused by disease. In addition, such limits are likely to benefit vision screening in visually demanding occupational environments when the applicants are required to have normal limits for spatial vision. In an aging population and extended working lives,^47^ it becomes more important to detect when the worsening of spatial vision exceeds normal age limits. In many western countries, the retirement age is increasing, and it is well‐known that older individuals need more light to carry out visual tasks, even when younger people find the same tasks relatively easy.^48^ Normal visual performance in the mesopic range is also important in safety‐critical occupational environments involving pilots, air traffic controllers, train drivers, seafarers, rapid response drivers, firearms officers and firefighters.^12^, ^49^, ^50^, ^51^ For example, in the Salisbury Eye Evaluation Study, luminance levels of 5.2 cd/m^2^ were used to investigate whether mesopic VA is a predictor for car crash involvement.^52^ A luminance level of 5.2 cd/m^2^, although considered low in this study, is higher than the working luminance levels encountered in a number of occupations. In addition, measurements under mesopic conditions are considered to be more sensitive in medical selection.^53^ It is therefore generally agreed that the assessment of both VA and CS should not be limited to only photopic vision but should also be measured at lower light levels when poorer performance can be indicative of impaired photon absorption efficiency in photoreceptors,^19^ and/or neural changes that precede retinal disease.^54^ Despite the obvious advantages of testing VA in the mesopic range and the availability of open source software to carry out VA and contrast sensitivity tests,^55^, ^56^ standard methods have not been developed and upper normal limits of spatial vision in the mesopic range have not been established.^7^, ^57^, ^58^ This makes screening for abnormal responses and the comparison of results from different studies difficult to carry out.

Binocular vision yields significant improvements in many aspects of vision, particularly in the mesopic range. A knowledge of binocular VA and CT limits of visual performance as a function of age is of great interest in occupational environments.^59^ In contrast, the early detection of ocular disease requires reliable normal, monocular, upper threshold limits for VA and contrast in order to screen for abnormal responses in each eye. In clinical practice, both VA and CTs are measured with negative contrast optotypes on illuminated test charts, i.e., black optotypes produced by depositing spectrally neutral pigments on a high reflectance, neutral background. Although differences in spatial vision between negative and positive contrast have been examined in previous studies,^60^ little has been done to produce standard methods for assessing spatial vision with both contrast polarities. Although negative contrast optotypes do not always yield lower contrast thresholds, the majority of studies report improved performance with negative contrast stimuli,^61^, ^62^ both in terms of VA and CT, and absolute detection thresholds when measured with decrements in luminance.^63^ When used in the clinic in patients with early‐stage retinal disease and high levels of scattered light, contrast polarity can produce unexpected results with higher thresholds corresponding to positive contrast optotypes.^64^, ^65^ Another parameter that affects the outcome of VA and CS tests, particularly in patients with loss of spatial vision as a result of early retinal disease, is the stimulus presentation time.^66^ Normal aging affects the temporal impulse response function of the eye with significant loss of the inhibitory phase of the impulse response in some older subjects and the subsequent loss of temporal sharpness and reduced response amplitude.^67^

This study employed optimized parameters for the assessment of spatial vision based on preliminary investigations and results from earlier studies reported in the literature.^13^ These parameters were then combined in a single test designed to assess monocular and binocular VA and CT with both positive and negative contrast optotypes at photopic and high mesopic retinal illuminance levels. In all, 16 different viewing conditions have been examined to obtain reliable, mean and upper and lower normal threshold limits for VA and contrast as a function of age to benefit the assessment of spatial vision, both within occupational health and in the clinic.

## METHODS

### Participants

A total of 382 participants with age range between 10 and 86 years were recruited at three different sites in the Netherlands: (1) a private eye clinic (Damme Optometrie in Kesteren); (2) a university eye clinic (University of Applied Sciences, Utrecht) and (3) a normal working environment (City Hall of Alphen aan den Rijn). The inclusion of a primary care setting, educational institution and workplace environment was a conscious choice to maximize random sampling in diverse populations. The study was approved by the Research and Ethics Committee at the City, University of London and the Medical Ethical Committee at the University Medical Centre, Utrecht, the Netherlands. All participants provided written consent. In cases where participants were younger than 16 years old, the consent form was, in accordance with Dutch law, signed by the participants' parents/legal guardians (10–11 years) or by the participant (child) and parents/legal guardians (12–15 years).

### Ophthalmic assessment

A detailed medical history was taken: all participants or parent/guardian answered questions about their general health, use of medications, ocular health and general and ocular family history. A full objective and subjective refraction were conducted at a test distance of 3 m. VA was then measured monocularly and binocularly in logMAR units with the 2000 series revised Early Treatment Diabetic Retinopathy Study (ETDRS) chart 2 (Precision Vision, precision‐vision.com) using the updated prescription. The original ETDRS illuminator cabinet was used to generate a chart luminance of 160 cd/m^2^. The 3 m distance was selected for convenience and to match the viewing distance employed in the Acuity‐*Plus* test. The anterior segment was assessed using a Topcon SL‐7F slit lamp (Topcon, topcon.com) or CSO SL9900 5X‐D (CSO Italia, csoitalia.it), and the transparency of the lens was noted for each participant and classified according to the Optometric Grading Scales.^68^ This scale consists of a set of drawings showing different lens opacities based on the Lens Opacities Classification System III (LOCS III) photographs.^68^ In order to minimize the interexaminer variability, all ophthalmic/clinical assessments were performed by the same examiner for all testing sites. The fundus was assessed with undilated, indirect ophthalmoscopy and photographed with a digital nonmydriatic retinal camera (Topcon TRC‐NW65 [Topcon, topcon.com]) or Canon CX‐1 (Canon, canon.com).

### Visual acuity and contrast sensitivity assessment with the Acuity‐*Plus* test

High contrast VA and CTs with optotypes of both positive and negative contrast were measured in each participant using the Acuity‐*Plus* test (City Occupational, city‐occupational.co.uk). This test of spatial vision is one of a series of Advanced Vision and Optometric Tests (AVOT) developed at the City, University of London for use in occupational health and in the clinic. The Acuity‐*Plus* test employs a stable, high‐resolution, 10‐bit dynamic range visual display (NEC spectraview 2690WU, sharpnecdisplays.com) which the participant views from a distance of 3 m. The room was darkened at all three locations and the low mesopic ambient lighting was attributed to the light produced by the operator's monitor and the stimulus background field on the visual display. This ensured that the ambient lighting remained very similar across the three testing sites. The testing display is fitted with a hood to minimize ambient lighting, and initial adjustments were carried out by the manufacturer to minimize the black light level and to achieve a maximum luminance of ~146 cd/m^2^ in native colour mode. Furthermore, the stimulus display was checked for luminance calibration periodically and recalibration of each primary colour was performed when required using the LMT‐1009 luminance meter (LMT, lmt.de) and the LUMCAL calibration program (LUMCAL, City Occupational, city‐occupational.co.uk). A high‐performance laptop drove the display via a VESA DisplayPort interface, which supports 10‐bit output graphics needed to match the dynamic range of the visual display. All participants performed the Acuity‐*Plus* test with full updated correction for the testing distance of 3 m, which was provided in a trial frame, to ensure that testing conditions for all participants were equivalent with respect to spectacle properties. The VA and CTs were measured using a four‐alternative, spatially‐aided, forced‐choice procedure based on four, randomly interleaved, two‐up/one‐down, staircases with variable step sizes. The chance probability of a correct response is 1/16 and the thresholds measured correspond to 71% probability of a correct response.^11^ The stimulus consisted of a Landolt C optotype with the gap positioned randomly in one of the four quadrants, and each test measured four parameters, i.e., VA and CTs with both positive and negative contrast. In all measurements, the participant had to detect and ‘register’ the location of the gap in the Landolt C optotype. A short beep at the end of each stimulus presentation prompted the participant to press one of the four raised buttons on the numeric keypad to report the perceived location of the gap. When unsure about the gap location, the participant's instruction was to guess the most likely location without hesitation. Both photopic and mesopic spatial vision was assessed using the standard mesopic and photopic protocols in the Acuity‐*Plus* test. Short breaks separated successive tests to minimize fatigue and the participants were also encouraged to take additional breaks whenever needed during the session. The mesopic protocol was always preceded by ~10 min of adaptation to the low luminance screen employed in the mesopic condition. For this protocol, the participants wore spectrally calibrated, ‘neutral density’ sunglasses (Oakley Garage Rock, oakley.com). The program employed the known spectral transmittance of the sunglasses to ensure that when viewed through the glasses, the stimulus display had the correct luminance and chromaticity. A ‘learning mode’ option preceded any measurements of VA or CTs. This brief test required 100% correct responses and ensured that every applicant was familiar with and understood the task. Every participant carried out the initial learning test under binocular viewing conditions. The order of testing (monocular, binocular) was randomized for both photopic and mesopic conditions. The study employed the Acuity*‐Plus* standard photopic and mesopic protocols. The standard photopic protocol measured VA and CTs at the fovea with both positive and negative contrast stimuli for a screen luminance of 32 cd/m^2^ and CIE—(x, y) chromaticity coordinates of 0.305, 0.323. The standard mesopic protocol measures the same four parameters using light of the same chromaticity but with a screen luminance of 2 cd/m^2^. The choice of adapting background luminance was based on typical luminances encountered in mesopic work environments when good spatial vision is still needed in order to carry out the visual tasks. Similar light levels are also found in many lit spaces at night and in traffic situations when safety remains an important requirement.

The stimulus was presented for 160 ms to avoid letter scanning and hence multiple fixations. When this presentation time is employed, the majority of normal trichromats achieved a spatial resolution better than 1 min of arc (1′) acuity, i.e., a Landolt ring size of 5′ outer diameter converts to an equivalent Snellen VA of 6/6 and a LogMAR VA of 0.0. The conversions are simple; LogMAR = log _10_ (MAR), where MAR is the size of the gap (in minutes of arc) needed at the threshold to locate its position. Alternatively, when using the most common Snellen notation, VA_Snellen_ ~ 6/(6 × MAR). This means that a Snellen VA of 6/6 corresponds to a MAR of 1′. Doubling this to 2′ makes the Snellen acuity 6/12. The VA results in this paper are presented in log units (LogMAR) and shown in all tables and graphs. A linear scale is also provided for convenience. CTs, measured in percentage contrast, are plotted on a log scale and the corresponding values are also given in log units.

### Selection of participants for inclusion in the study

**FIGURE 1:**
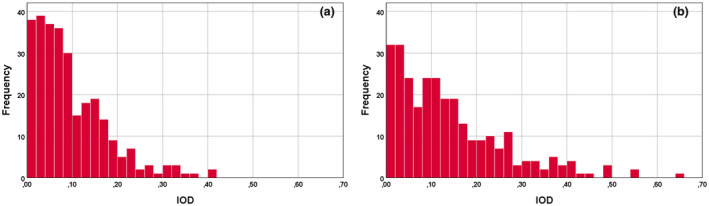
Frequency histograms showing examples of observed distributions of fractional differences between the two eyes for photopic visual acuity (VA) measured with negative contrast (a) and for photopic contrast thresholds (CTs) measured with positive contrast (b). The measured variables were converted to log units, and the interocular difference (IOD) is expressed as IOD = ABS(T_RE_ − T_LE_), where T_RE_ and T_LE_ represent the thresholds measured for each stimulus condition in the right and the left eyes, respectively, in log units. The mean values for T_RE_ − T_LE_ are close to zero, but the use of absolute values for the differences in the measured thresholds in the two eyes doubles the number of measurements on one side of the histogram. Participants with absolute thresholds greater than 2.5σ are not included in the analysis.

The principal aim of this study was to establish mean values and upper normal limits of spatial vision as a function of age. In order to achieve this aim, a number of filters were employed to ensure that the participants included in the study had ‘normal visual performance’ for the corresponding age. Each of the included participants fulfilled the following requirements:

- Absence of a medical history of ocular or systemic conditions known to affect vision. Participants were arranged into groups by type of chronic condition. *t*‐test analyses were performed to determine significant differences between participants with and without each of the selected conditions. If there was no difference between the selected chronic condition subgroup and the remaining subjects with no such conditions, the participants were included in the study. This was the case for participants with hypertension who rarely exhibit significant loss of spatial vision. However, those diagnosed with diabetes were excluded since this systemic condition is known to affect several aspects of vision.^69^, ^70^, ^71^, ^72^, ^73^
- Absence of current signs of ocular disease, conventional or refractive laser surgery, corneal dystrophies or lens extraction. Participants with nuclear, cortical and posterior subcapsular lens opacities graded 3 or higher (according to the Optometry Grading Scale) were excluded.^68^ The remaining participants were included in our sample simply because a grading of two or lower is very common in an aging population and, as a result, one may be justified to attribute these smaller changes to ‘normal’ healthy aging.
- A new filter was developed and applied to detect those with potential subclinical, but yet unidentified, visual problems. The filter relies on the comparison of thresholds measured with the same stimulus parameters in each of the two eyes. Since changes caused by either the optics of the eye or diseases of the retina rarely affect both eyes in exactly the same way, participants with abnormal differences in VA and/or CTs between the two eyes were not included in the analysis for normal age limits. The index employed to describe the interocular difference (IOD) between the log values of the measured thresholds in the two eyes was, IOD = ABS(Log RE − Log LE), with RE and LE referring to the right and left eyes, respectively. Figure 1 shows the statistical distribution of this parameter for both VA (with negative contrast optotypes) and for CTs (with positive contrast) when using the photopic protocol. Similar graphs were obtained for the remaining 14 stimulus conditions. All participants with threshold differences greater than 2.5σ units were classed as outliers and excluded from the analysis.
- In addition, the results for the remaining study participants were reanalysed per decade and all outliers with log thresholds outside the ±2.5σ range with respect to the corresponding mean threshold values were removed from the analysis. This filter was applied separately to each measurement condition. At this last stage of screening for normal healthy vision, the study participants eliminated from the analysis varied from 0% to just under 2.5%, depending on the stimulus condition.

### Statistical analysis

Statistical analyses were carried out using SPSS (version 25, IBM, ibm.com) and JMP (version 14, jmp.com). Distributions and frequencies of results for categorical variables were examined in each decade. Skewness, kurtosis and Shapiro–Wilk were conducted as normality tests. The filters described made it possible to identify those participants who failed to perform the spatial vision tasks within the expected, normal statistical limits for the corresponding age. Paired samples *t*‐tests were used to compute differences between the positive and negative contrast measurements per decade, and a one‐way analysis of variance (ANOVA) was conducted to compare the photopic and mesopic measurements between the different test locations.

Participants were initially separated into decades during recruitment and analysis. The aging trend was minimal within decades and the statistical estimates based on the number of participants examined per decade were more reliable because of larger sample sizes. This approach is in agreement with other studies^35^, ^43^, ^74^ on the effects of age on visual function.^75^ Fitting nonlinear functions to mean and ± 2.5σ limits produced better results with smaller range limits for the best‐fit parameters when more points were involved. The number of points was therefore doubled to benefit the Gauss–Newton, nonlinear, curve‐fitting method by using 5‐year bins. The calculated threshold limits for each subgroup correspond to the mean, mean +2.5σ (Upper Normal Limit, UNL) and mean −2.5σ (Lower Normal Limit, LNL). A model with biologically meaningful parameters was fitted to the data to predict LNL, mean and UNL functions for each of the 16 data sets. Preliminary examination of the data helped with the selection of the starting values for the model parameters. Thresholds were stable or increased minimally in the first few decades, but exhibited a more rapid increase in both mean values and intersubject variability above 50 years of age in photopic conditions and above 30 years of age in mesopic conditions. The following, four‐parameter, nonlinear model was fitted to each of the 16 sets of data investigated:

(1) $$\text{Dependent variable}=b_{1}+b_{2}\times \left\{\mathrm{Exp}\;{\left(\mathrm{Age}-b_{3}\right)}^{\text{b4}}-1\right\}$$

This data‐inspired model allows us to attach some meaning to describe the observed characteristics of normal healthy aging of spatial vision:

- b_1_ is largely determined by the upper horizontal asymptote when age has little, if any effect, on the measured thresholds,
- b_2_ is a weighting factor that applies to every age but only affects the results significantly when the participant's age is greater than b_3_,
- b_3_ is an important parameter, which determines the age above which the exponential function starts affecting the measured thresholds and is followed by a more increase in threshold with increasing age.
- Finally, parameter, b_4_, controls the speed of exponential growth in thresholds with advancing age.

The fitted curves are plotted as a function of age together with the measured thresholds for each of the study participants. The best‐fit model parameters, which describe the mean and the lower and upper normal threshold limits, are also listed in each graph.

## RESULTS

### Study population

**FIGURE 2:**
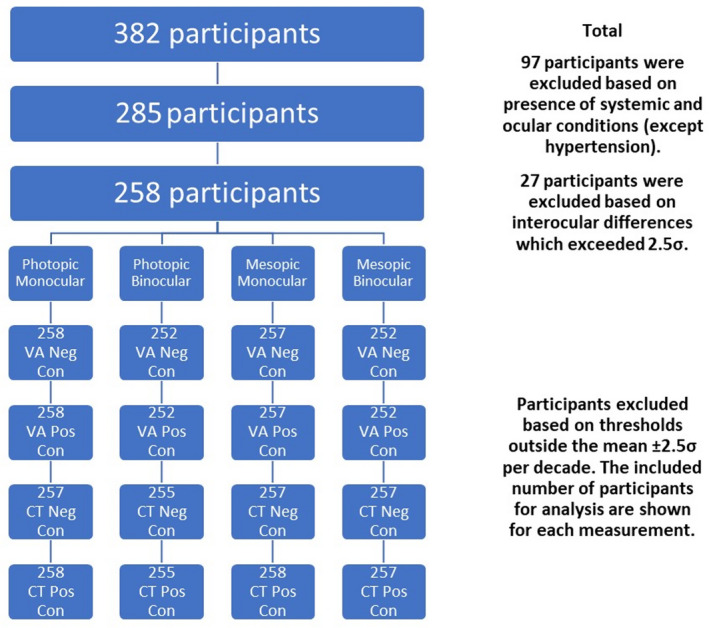
Flowchart showing the number of participants who failed each of the filtering criteria employed in the study. The very small differences in the final sample sizes are caused by applying the 2.5σ filter separately to each of the 16 stimulus conditions. con, contrast; CT, contrast threshold; neg, negative; pos, positive; VA, visual acuity.

Three hundred and eighty‐two participants were enrolled in this study. Independent *t*‐tests revealed no statistical difference between participants with hypertension and age‐matched healthy participants (*p* > 0.004). Hypertension was therefore not used as an exclusion criterion. The subgroups with other systemic and ocular diseases were too small to analyse reliably and were therefore excluded from the normal group. The effects of applying the various filters to the study population are illustrated in Figure 2. Because of applying the filters described above, the study involved between 252 and 258 participants depending on the stimulus condition employed. Table 1 lists the baseline characteristics of the participants included before the filter per condition was applied.

**TABLE 1:** Baseline characteristics of participants

	Participants (*n* = 258)
Age (years; M [SD])	43.4 (19.3)
Gender	
Male *n* (%)	103 (39.9)
Female *n* (%)	155 (60.1)
Spherical equivalent refractive error RE	
Myopic *n* (%)	144 (55.8)
Hyperopic *n* (%)	72 (27.9)
Emmetropic *n* (%)	42 (16.3)
Spherical equivalent refractive error LE	
Myopic *n* (%)	141 (54.7)
Hyperopic *n* (%)	71 (27.5)
Emmetropic *n* (%)	46 (17.8)
Ocular lens opacities: optometry grading scale RE	
Cortical M (SD)	0.04 (0.26)
Nuclear M (SD)	1.00 (0.83)
Posterior subcapsular M (SD)	0.02 (0.17)
Ocular lens opacities: optometry grading scale LE	
Cortical M (SD)	0.03 (0.23)
Nuclear M (SD)	1.00 (0.83)
Posterior subcapsular M (SD)	0.02 (0.13)
ETDRS photopic VA in logMAR	
RE M (SD) (M in MOA)	−0.09 ± 0.09 (0.84)
LE M (SD) (M in MOA)	−0.09 ± 0.09 (0.83)
Binocular M (SD) (M in MOA)	−0.15 ± 0.08 (0.72)

Abbreviations: ETDRS, Early Treatment Diabetic Retinopathy Study; LE, left eye; logMAR, logarithm of the minimum angle of resolution; M, mean; MOA, minutes of arc; *N*, number; RE, right eye; SD, standard deviation; VA, visual acuity.

### Visual acuity and functional contrast sensitivity outcomes

Table S1 lists the mean VA and CT results in log units measured with the Acuity‐*Plus* test for both the photopic and the mesopic conditions. Paired *t*‐tests were carried out to assess significant differences between the two eyes. All statistical comparisons carried out employed Bonferroni correction to account for multiple comparisons. No significant differences were found between the right and left eye VA and CT results (*p* > 0.01). The order in which the two eyes were tested had no significant difference in the results for both VA and CTs (*p* > 0.01). Within the same eye, the measured differences between positive and negative contrast optotypes were found to be statistically significant for all VA and CT data sets (*p* < 0.008). Overall, lower thresholds for both VA and CT were obtained with negative contrast. Photopic VA thresholds measured with negative contrast optotypes were also compared against the equivalent ETDRS VA data. Paired *t*‐tests revealed significant differences between the two tests with slightly larger VA thresholds when measured with the Acuity*‐Plus* test (*p* < 0.02). A one‐way ANOVA was conducted to compare the photopic and mesopic measurements between the testing sites. There were no statistically significant differences per decade between groups for all measurements as determined by one‐way ANOVA (*p* > 0.004).

**FIGURE 3:**
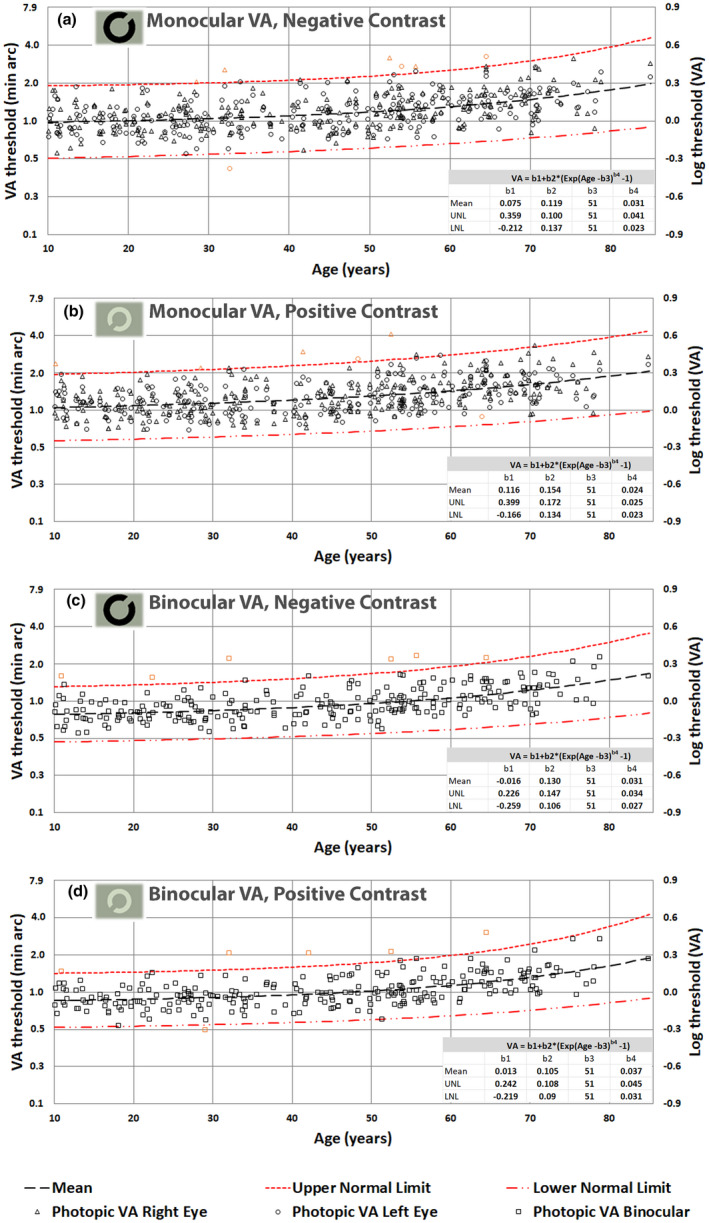
(a–d) Photopic visual acuity (VA) thresholds in minutes of arc and the corresponding LogMAR units plotted as a function of age; monocular negative contrast (a), monocular positive contrast (b), binocular negative contrast (c) and binocular positive contrast (d). The inset for each stimulus condition lists the parameters needed to predict the fitted functions (i.e., dependent variable = b_1_ + b_2_ × {exp (age − b_3_)^b4^ − 1}).

**FIGURE 4:**
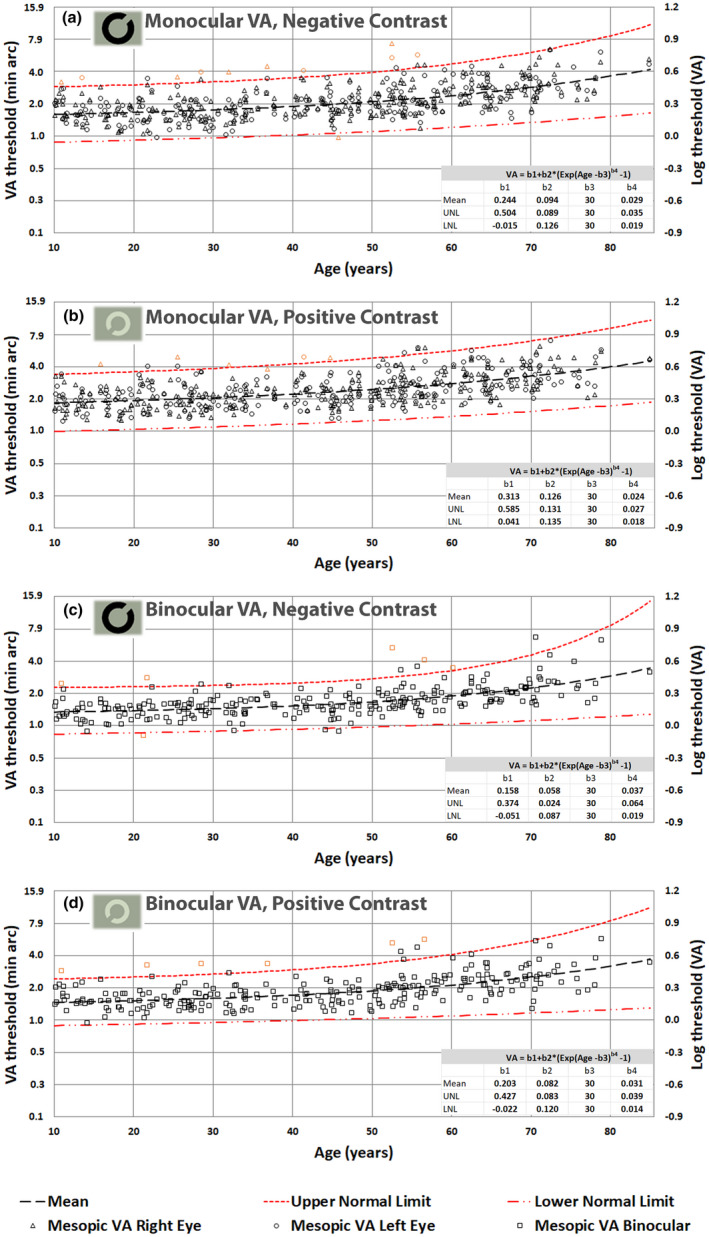
(a–d) Mesopic visual acuity (VA) thresholds in minutes of arc and the corresponding LogMAR units plotted as a function of age; monocular negative contrast (a), monocular positive contrast (b), binocular negative contrast (c) and binocular positive contrast (d).

**FIGURE 5:**
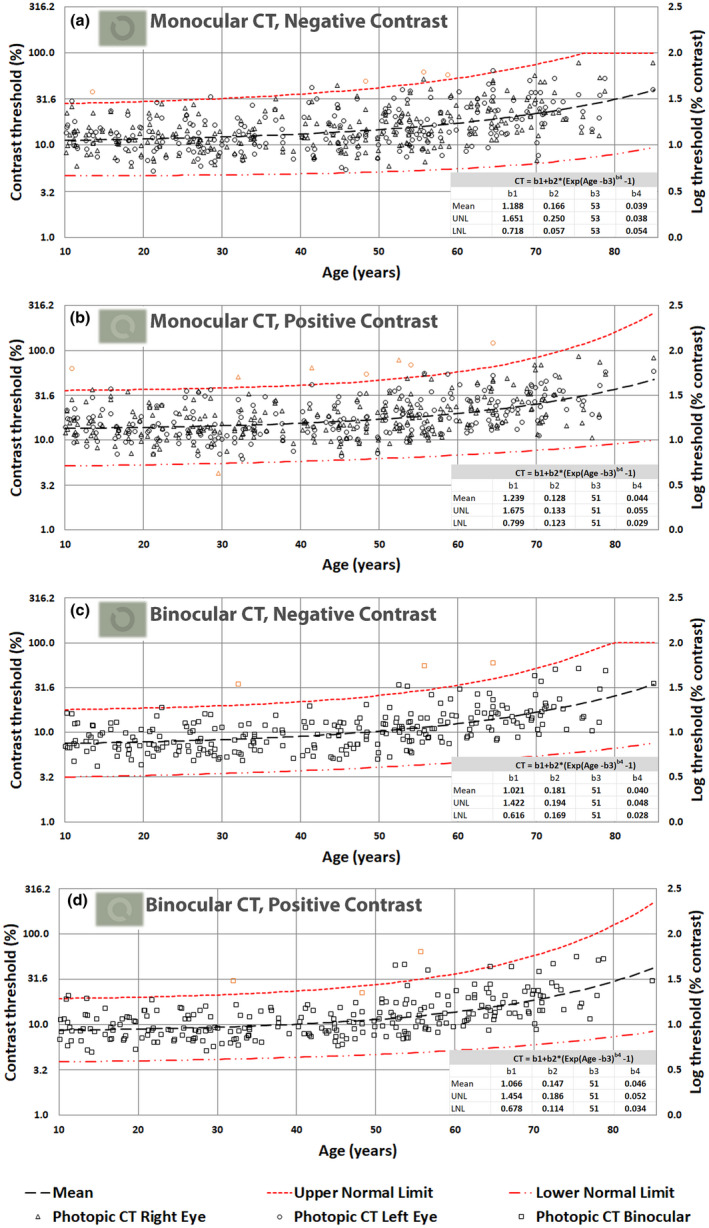
(a–d) Photopic contrast thresholds (CTs) in percentage luminance contrast and the corresponding values in log units, plotted as a function of age; monocular negative contrast (a), monocular positive contrast (b), binocular negative contrast (c) and binocular positive contrast (d).

**FIGURE 6:**
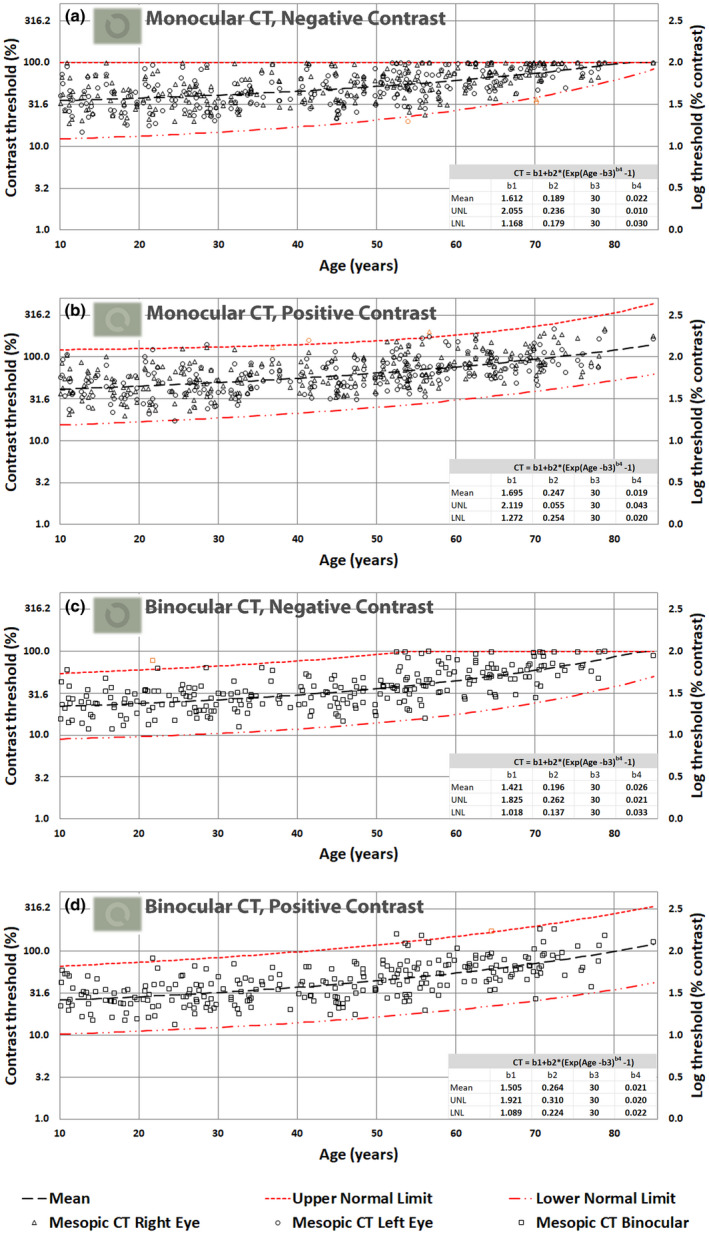
(a–d) Mesopic contrast thresholds (CTs) in percentage luminance contrast and the corresponding log units, plotted as a function of age; monocular negative contrast (a), monocular positive contrast (b), binocular negative contrast (c) and binocular positive contrast (d). Since the maximum negative contrast of single optotypes cannot exceed 100%, some subjects cannot resolve the 3′ gap size, even when presented at maximum contrast (see sections a and c). These results illustrate the large intersubject variability in contrast thresholds in the mesopic range. A few of the younger subjects had some difficulty with this task, even at 100% contrast (section a), but the majority of subjects above 60 years of age could not do the task. Consequently, upper normal limit (UNL) thresholds of 100% plotted in sections a and c simply indicate that the subjects were unable to detect the gap at 100% contrast. As a result, the mean values will also be affected and the UNL was limited by the maximum negative contrast one can generate on the visual display.

Figures 3a–d, 4a–d, 5a–d and 6a–d display the thresholds for each study participant measured in each of the 16 experimental conditions investigated in this study. The VA thresholds are presented both in log units (i.e., LogMAR) and in minutes of arc. Similarly, CTs are shown as Log (% contrast threshold) and percentage contrast. The outliers based on the 2.5σ filter are also plotted in orange symbols on each graph. In addition, each figure also plots the predictions of the model for the mean VA and CT as a function of age, together with the corresponding predictions for upper and lower normal threshold limits. The best‐fit model parameters are needed to predict mean values, and the upper and lower normal limits as a function of age are included in each graph.

Mean photopic thresholds and overall variability remain largely invariant below 50 years of age. Above 50 years, both mean thresholds and the observed intersubject variability increase with age (Figures 3a–d and 5a–d).

In the mesopic conditions, both VA and CTs start with much larger values (e.g., parameter b1 in the fitted model) and the thresholds start to increase more rapidly above 30 years of age, particularly for optotypes of positive contrast. The fitted curves capture well the observed effects of normal aging for both photopic and mesopic conditions. Independent *t*‐tests reveal significant differences (*p* < 0.004) between mean thresholds per decade and the mean threshold for the subsequent decade only when above 50 years of age.

## DISCUSSION

The principal aim of this study was to establish how the healthy normal aging of the eye and visual pathways affect spatial vision under photopic and high mesopic lighting conditions. To achieve this aim, we needed a sensitive and efficient test of spatial vision to measure VA and CTs at photopic and high mesopic light levels. The Acuity*‐Plus* test fulfils many of the requirements of this study. The test was initially designed to assess the effects of corneal refractive surgery on visual performance under photopic and mesopic lighting,^12^ and has more recently undergone improvements in stimulus parameters and methodology. Each standard protocol measures four parameters of interest, which provide useful information on the subject's spatial vision. The interleaved measurement of these parameters minimizes the effects of other factors such as fatigue and variations in pupil size. Our aim was to recruit a random sample of participants ranging from 10 to 90 years and to examine each participant to ensure fulfilment of the criteria for acceptance into the study. This was particularly challenging for subjects above 70 years of age. By testing each eye separately, we were able to identify 27 outliers as defined by statistically significant differences between the two eyes. The need to fulfil the exclusion criteria illustrated in Figure 2 ensured that the subjects selected for the study exhibited only gradual changes to the optics of the eye and the visual pathways that are commonly found in the normal population and can be attributed to the innumerable changes that affect the visual system in normal aging. The filters applied prevented the inclusion of participants with known ocular conditions that affect spatial vision. It has been reported that impaired mesopic acuity in clinically healthy eyes can precede age‐related macular degeneration (AMD),^54^ and was also found in carriers of AMD risk genotypes.^21^ The analysis of the large data set aimed to produce mean, lower and upper normal limits as a function of age for each of the 16 test conditions. An equation with four meaningful parameters was fitted to each set of data to allow for the prediction of VA and CTs for any age for each of the stimulus conditions investigated. The log‐transformed data measured for the majority of the tests carried out were found to be normally distributed, and this allowed us to use mean values and parametric tests for within and intersubject comparisons. Some results, particularly those measured in older subjects using the mesopic conditions produced more residual skewness. Since these older participants passed the filtering conditions designed to screen for normal healthy vision, residual skewness of the data observed above 70 years of age is taken to reflect normal aging. This, and the observed increase in intersubject variability with increasing age, required the use of a nonlinear model. The model defined by Equation 1 was fitted to each set of data points to predict mean values and ±2.5σ limits. These functions describe normal gaining according to the selection criteria employed in the study. The b1 parameter reflects mean threshold values expected in young subjects before the effects of age become significant. Higher values for the b3 parameters, which vary around 50 years of age in Figures 3a–d and 5a–d and around 30 years of age in Figures 4a–d and 6a–d are indicative of the age above which the measured thresholds start to increase more rapidly. The higher rate of exponential increase becomes noticeable above 60 years of age and continues with increasing age, although extrapolation above 80 years of age is less justified because of fewer data points and potentially larger intersubject variability.

The largest thresholds were measured with optotypes of positive contrast and correspond to the monocular viewing condition. The best performance for both VA and CTs is achieved with negative contrast optotypes in binocular viewing. VA and CTs in the photopic range appear to be stable up to 50 years of age. These results are in general consistent with findings from other studies.^29^, ^36^, ^37^

Both mean thresholds and intersubject variability are reduced significantly in binocular viewing, and this was particularly evident for both VA and CTs in the mesopic range. The majority of participants exhibit large binocular summation, which is in agreement with earlier findings.^13^, ^76^, ^77^, ^78^ Despite the large improvement in mesopic thresholds in binocular viewing, both VA and CTs remain significantly worse when compared to equivalent results in the photopic range.

The choice of 2 cd/m^2^ for use in the Acuity‐*Plus* protocol is consistent with typical residential street lighting and other mesopic working environments where adequate spatial vision is required.^52^, ^79^, ^80^ Light levels below 0.2 cd/m^2^ are considered to be more representative of the mesopic range,^28^ but less representative of working environments which rarely fall below 2 cd/m^2^ and also less useful clinically because of the much increased within and intersubject variabilities. The largest variability in both VA and CTs corresponds to monocular measurements with positive contrast optotypes and hides the significant increases in mean thresholds during the first few decades of life.

The results show that even in the high mesopic range, both VA and CTs are more susceptible to aging than the corresponding findings in the photopic range. A number of different factors may contribute to the more rapid worsening of spatial vision with increasing age in the mesopic range. Increased pupil miosis and greater absorption and scattering of light by the lens cause a significant reduction in retinal illuminance, and this results in a greater loss in retinal sensitivity to contrast in older subjects in the upper mesopic range.^27^, ^28^ Increased higher order aberrations and forward scatter in the eye cause decreased retinal image contrast and hence a reduction in VA and CS with advancing age.^32^, ^36^, ^44^ In addition, the slight decrease in cone photoreceptor density and the gradual loss of ganglion cells may also contribute to the worsening of spatial vision.^81^, ^82^, ^83^, ^84^ The normal limits for VA and CTs derived in this study can be used to identify subjects with parameters that fall outside the normal age limits. It is not uncommon for an eye with increased higher order aberrations and scattered light to produce VA values outside the normal range and CTs measured with larger stimuli well within the normal range. This is simply because the larger stimuli employed in FCS tests are less affected by higher order aberrations and forward light scatter in the eye. The best CTs one can achieve with larger stimuli are often limited by retinal sensitivity to contrast. Normal VA involves the use of much smaller stimuli that are more affected by both aberrations and scattered light.^85^, ^86^ The opposite case also occurs when normal VA is accompanied by higher CTs. Such an outcome is consistent with good retinal image quality, but poor retinal sensitivity to contrast. Both VA and CTs are affected by the quality of the retinal image, the level of retinal illuminance and the normal functioning of the retina.

The results measured with negative contrast optotypes were significantly better than the corresponding thresholds measured with positive contrast, in agreement with findings from earlier studies.^61^, ^62^ The most pronounced differences were found at lower light levels in older participants. In contrast, measurements with the FrACT test,^55^, ^56^ which also employs Landolt ring stimuli, found no significant differences in photopic and scotopic VA between negative and positive contrast in young observers.^87^ This may be due to the smaller sample size and to the specific stimulus conditions of the study. The availability of open source software is attractive and may make it possible to adjust the parameters of the test to approximate those employed in our study. Should this be the case, the use of fixed parameters that are similar to those employed in the Acuity*‐Plus* test may well yield similar limits to those reported here. If so, then the use of the spatial limits obtained in this study that describe the effects of aging under standardized conditions could be extended to other tests. The normal age limits reported here are described fully, and equations are provided for each of the 16 stimulus viewing conditions. This makes it possible to compare our limits to those obtained with other instruments, in addition to the FrACT test. However, the validation studies may not be without challenges since the Acuity*‐Plus* test employs a fully calibrated 10bit display and spectrally calibrated ND glasses for use in the mesopic protocol. Although this approach is of value in order to achieve standardized conditions, we acknowledge that in terms of general use, the more expensive calibrated equipment and the much higher dynamic range may limit the availability of the test. Although not included in this study, similar measurements carried out in patients with diabetes and other ocular conditions reveal much larger differences in both VA and CTs when comparing results measured with equivalent optotypes of opposite luminance contrast. The combination of the four measurements into one single test has other significant advantages. In previous studies, VA and sensitivity to contrast were assessed using different test charts and in different experimental sessions.^88^, ^89^ As a result, no standardized methods for measuring spatial vision using similar stimuli for both photopic and mesopic conditions have been produced. The choice of different parameters in different tests, such as the size of the optotypes and the luminance and size of the adapting visual field make the comparison of results difficult and limit the usefulness of such measurements. In this study, we placed great emphasis on justifying the choice of parameters for photopic and high mesopic conditions with direct reference to vision requirements, both within occupations and in the clinic. Another important parameter in the Acuity‐*Plus* test is the stimulus presentation time of ~160 ms. This short time eliminates multiple fixations and minimizes within‐subject variability. In general, the use of a short presentation time results in slightly higher VA thresholds when compared to the same measurements under continuous viewing on ETDRS test charts.^66^ The processing of clear edges and contours during the brief presentation of the test stimulus requires normal temporal responses. Although the majority of participants with normal vision are minimally affected by the short stimulus presentation time with VA better than 1′ (logMAR 0.0, see Figure 3c), older participants tend to be affected more, particularly at lower light levels. The brief presentation time may make the test more sensitive when screening for early‐stage ocular diseases, for example, AMD. Such patients require much longer times to achieve the best acuity compared with age‐matched, healthy individuals.^90^ Longer presentation times also result in multiple fixations and this can aid in the self‐selection of the least‐affected retinal area that yields the highest sensitivity. However, recent studies have shown that the temporal impulse response of the eye broadens and is less able to reproduce sharp temporal edges in older subjects.^18^, ^67^ Significant loss of temporal responses has also been reported in patients with diabetes, glaucoma or AMD.^90^ Since the effective spatial contrast of a briefly presented stimulus is affected strongly by the temporal response function of the eye, it is not surprising that when the latter is reduced (either because of normal aging or disease), a high contrast, briefly presented stimulus is often equivalent to a continuously presented stimulus of lower contrast. Although the Acuity‐*Plus* test measures VA and CTs, the measured parameters are also sensitive to changes in the temporal response characteristics of the retina. A limitation of this study may be the selection of the study population in three different settings. This choice has, however, ensured the exposure of study participants to a variety of occupations and work‐related visual tasks. This can also be considered important when establishing normal age‐related limits for occupational use. Furthermore, all participants were Caucasian and hence the use of the limits derived from this study with other ethnicities rests on the assumption that any differences in spatial vision are small. Uncorrected refractive errors and astigmatism in particular have been shown to affect VA and CS.^91^, ^92^, ^93^, ^94^ In this study, each participant was refracted and corrected for the testing distance of 3 m. Residual, uncorrected refractive errors are therefore unlikely to have contributed significantly to the results.

Currently, visual function testing is often limited to photopic high contrast VA, simply because measures of CS are too demanding and require the investigation of several parameters using sinusoidal gratings, making the test often too long, complex and impractical in clinical practice.^95^ The Acuity‐*Plus* test is simple to carry out and the availability of upper normal age limits for each of the four measured parameters makes this assessment more valuable as part of the standard optometric examination. The measure of CTs introduced and investigated in this study relies on the measurement of only one luminance contrast for a fixed stimulus size. Since visually demanding tasks rarely employ alphanumeric characters smaller than three times the average acuity limit (i.e., 3 × 5′), the Landolt ring employed in this contrast threshold test was selected to have an outer diameter of 15′ with a 3′ gap size. The ability to resolve and locate a 3′ gap size in low contrast is functionally important in many visual tasks. The combined assessment of VA and CTs using photopic and high mesopic light levels with optotypes of positive and negative contrast provides a better description of the participant's spatial vision. Combining the four parameters and the availability of normal age limits can help in the early detection of retinal disease and may justify the use of the test in clinical practice. In particular, the mesopic measurements and the upper normal age limits established in this study may be of interest in the early detection of retinal disease.^54^ The standardized measurements can also be used to monitor the progression of ocular disease and to clarify patients' complaints in daily life activities under different lighting conditions. In addition, the normal VA and CT age limits are also useful in clinical trials by eliminating the need for age‐matched controls.

In conclusion, this study establishes upper, normal age limits for monocular and binocular viewing under photopic and high mesopic lighting with both positive and negative contrast optotypes using a single, efficient test that can be used in many occupational settings and in the clinic.

## AUTHOR CONTRIBUTIONS

**Arjan Keuken:** Conceptualization (equal); data curation (lead); formal analysis (lead); investigation (lead); methodology (lead); project administration (equal); resources (equal); validation (equal); writing – original draft (lead); writing – review and editing (equal). **Ahalya Subramanian:** Conceptualization (equal); methodology (equal); supervision (equal); validation (equal); writing – review and editing (equal). **Sigrid Mueller‐Schotte:** Formal analysis (equal); methodology (equal); supervision (equal); validation (equal); writing – review and editing (equal). **John L. Barbur:** Conceptualization (equal); formal analysis (equal); methodology (equal); resources (equal); supervision (equal); validation (equal); writing – review and editing (equal).

## CONFLICT OF INTEREST

John Barbur is the inventor of the Advanced Vision Optometric Tests (AVOT), some employed in this study; an employee of City, University of London; and a director of City Occupational Ltd. (a spin out company of City, University of London). Other authors: None.

## Supporting information


Table S1
