# Supplementary material for: Age‐related normal limits for spatial vision
Source: Ophthalmic Physiol Opt. 2022 Aug 18;42(6):1363–78. doi: 10.1111/opo.13037 (PMC9805202; doi:10.1111/opo.13037)
Supplement: Supplementary file 1 — Table S1 [file OPO-42-1363-s001.docx]

|  | Photopic condition | Mesopic condition |
| --- | --- | --- |
| Monocular assessment VA | **M LogMAR ± SD**  **(MOA)** | **M LogMAR ± SD**  **(MOA)** |
| Negative contrast RE | 0.07 ± 0.13 (1.18) | 0.32 ± 0.15 (2.09) |
| Positive contrast RE | 0.11 ± 0.14 (1.29) | 0.38 ± 0.15 (2.40) |
| Negative contrast LE | 0.06 ± 0.13 (1.15) | 0.31 ± 0.14 (2.04) |
| Positive contrast LE | 0.10 ± 0.13 (1.26) | 0.38 ± 0.15 (2.40) |
| Binocular assessment VA | **M LogMAR ± SD**  **(MOA)** | **M LogMAR ± SD**  **(MOA)** |
| Negative contrast | -0.02 ± 0.12 (0.96) | 0.22 ± 0.13 (1.66) |
| Positive contrast | 0.01 ± 0.12 (1.02) | 0.27 ± 0.14 (1.86) |
| Monocular assessment CT | **M LogCT ± SD**  **(PCT)** | **M LogCT ± SD**  **(PCT)** |
| Negative contrast RE | 1.18 ± 0.22 (15.14) | 1.71 ± 0.20 (51.29) |
| Positive contrast RE | 1.24 ± 0.21 (17.38) | 1.80 ± 0.21 (63.10) |
| Negative contrast LE | 1.17 ± 0.22 (14.79) | 1.69± 0.20 (48.98) |
| Positive contrast LE | 1.24 ± 0.21 (17.38) | 1.78 ± 0.21 (60.26) |
| Binocular assessment CT | **M LogCT ± SD**  **(PCT)** | **M LogCT ± SD**  **(PCT)** |
| Negative contrast | 1.02 ± 0.21 (10.47) | 1.54 ± 0.22 (34.67) |
| Positive contrast | 1.07 ± 0.21 (11.75) | 1.62 ± 0.23 (41.69) |
